# Histomorphological Description of the Digestive System of Pebbly Fish,* Alestes baremoze* (Joannis, 1835)

**DOI:** 10.1155/2017/8591249

**Published:** 2017-07-17

**Authors:** Nasser Kasozi, Gerald Iwe Degu, Julius Mukalazi, Charles Drago Kato, Majid Kisekka, Akisoferi Owori Wadunde, Godfrey Kityo, Victoria Tibenda Namulawa

**Affiliations:** ^1^Abi Zonal Agricultural Research & Development Institute, National Agricultural Research Organisation, P.O. Box 219, Arua, Uganda; ^2^College of Veterinary Medicine, Animal Resources & Biosecurity, Makerere University, P.O. Box 7062, Kampala, Uganda; ^3^Aquaculture Research & Development Center, National Agricultural Research Organisation, P.O. Box 530, Kampala, Uganda

## Abstract

Histomorphological studies of the digestive system of* Alestes baremoze *captured from Lake Albert, Uganda, were done using standard procedures. These revealed that* A. baremoze *has a fleshy-lipped terminal small mouth, large molar, short oesophagus, a three-lobed liver, pouch-like stomach, a nine-fingered caeca, and a long tubular intestine. A stratified squamous epithelium with numerous mucus-secreting cells lined the lips with no taste buds. Stratified squamous epithelia lined the oesophagus in the anterior portion which turned into a columnar epithelium towards the stomach. The lamina propria had numerous tubular glands throughout the entire oesophageal length. The stomach consisted of three distinct regions (cardiac, fundic, and pyloric) with distinguished lamina propria glands. The intestinal mucosa was thrown into villi of varying heights, with the tallest in the anterior part, lined with a simple columnar epithelium with numerous lymphocytes-like infiltrations. Numerous goblet cells appeared in the intestinal lamina epithelialis; these increased uniformly towards the anal opening. The liver was divided into lobules, with a central vein. Hepatocytes were visibly arranged closely, forming irregular cords, and the scattered tubular acinar glands formed the exocrine pancreas (hepatopancreas). Stomach content analysis indicated that the fish eats plankton, mollusks, crustaceans, and insects as the main proportion of its diet.

## 1. Introduction

*Alestes baremoze* (Joannis, 1835) or Pebbly fish is native to freshwater systems in Africa, thriving well in both lacustrine and riverine conditions. It belongs to order Characiformes, family Alestidae, and genus* Alestes* [1]. In East Africa, the fish appears in Lake Albert, Lake Turkana, and the Albert and Murchison Niles. In Northeast Africa, the species is present in the Ghazal and Jebel systems, White and Blue Niles in Sudan, and the River Nile, as far as Lake Nasser. In West Africa, it appears in Chad, Niger/Benue, Volta, Comoé, and Bandama [1]. In Uganda,* Alestes baremoze* is a delicacy and a high value fish species particularly in the West-Nile region, where it is also popularly known as “*Angara*” [2, 3]. Studies indicate that the fish has been extensively harvested, putting it under threat [1]. Similarly, recent research indicates tremendous species decline [2, 4]. The fact that* Alestes baremoze* is increasingly getting scare, yet greatly demanded on the market, makes it one of the species worth considering for commercial aquaculture. Despite the vast studies conducted to understand the feeding ecology of this fish in Lake Albert [5, 6], no information is available on the anatomy of its digestive system.

Insights into the anatomy of the digestive system of an aquaculture candidate fish are of importance in guiding development of appropriate feeding strategies of the fish once cultured [7, 8]. The structure of fish gut is a complex system [9], responsible for food ingestion, digestion, and assimilation [10], and the way this is done depends on the anatomical design of the digestive system [11]. For this reason, several studies have been done to describe the structure of the digestive system in different fish species [12–15]. Similarly, several research teams have analysed the relative gut length (RGL) in fishes and their feeding regimes [16–18]. However, no such investigation has been done in* A. baremoze*, and this limits the understanding of the functioning of the digestive system in this fish and its diet, yet such information is important to guide the development of feeding strategies in farmed fish. The aim of this study therefore was to describe the histomorphology of the digestive system of* A. baremoze* and its RGL and stomach content so as to gain insights into the functioning of this system; such information will guide the development of appropriate feeding strategies for this fish once cultured.

## 2. Materials and Methods

### 2.1. Sample Collection

**Figure 1:**
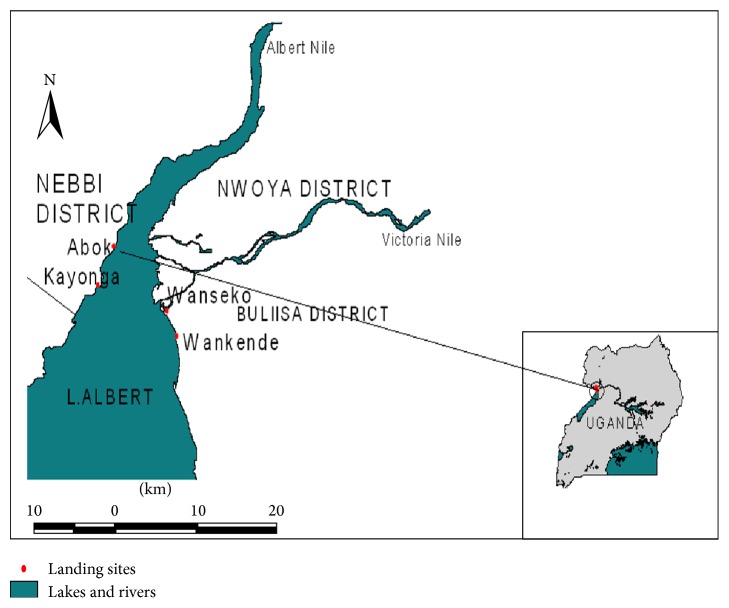
Map showing the study site.

A total of 20 samples of males and females of adult* A. baremoze* were caught using gill nets of 4 inches from Lake Albert and landed at Abok fish landing site (02° 14.46′N 31°19.15′E) located in Panyimur subcounty on Lake Albert (Figure 1). The fish samples had a mean fork length of 40.7 (±1.47) cm and mean body weight of 1052 (±277) g. The fresh samples were dissected to expose the entire digestive system. The topography and gross anatomy of the digestive system were then described following successful dissection of the individual organs. At each dissection step, photographs of both the fixed and fresh samples were taken using a digital camera (Kodak EasyShare C763-7.1 megapixels).

### 2.2. Histological Description

Tissue sections comprising four of each of the different digestive system sections (oral cavity, oesophagus, stomach, caeca, intestine, and liver) were instantly fixed in Bouin's solution. Standard histological procedures [16] were used in tissue processing. The prepared tissue sections were stained using haematoxylin and eosin (H&E) and Masson's trichrome staining protocol [17]. The stained sections were then visualised under a Carl Zeiss (German) light microscope mounted with a 10-megapixel digital camera (Canon PowerShot A640, China). Microphotographs were captured and analysed using the ZoomBrowser EX photographic software (version 2.0).

### 2.3. Relative Gut Length

Gut lengths (cm) and total body lengths (cm) were obtained from 90 samples collected over a period of nine months (pooled samples of males and females). The values of relative gut indices of adult* A. baremoze* were studied [18] using the following formula: relative gut length = gut length [cm]/standard body length [cm].

### 2.4. Stomach Content Analysis

The contents of the stomach were collected separately and preserved in 70% ethanol before transportation to Makerere University for analysis. The stomach contents were identified under a light microscope (10–100x) and analysed using point methods [18, 19]. Food items were awarded points based on the volumes they were judged to have occupied. Stomach fullness was assessed on a 0–100-point scale with 0, 25, 50, 75, and 100 representing empty stomach, quarter full, half full, three-quarter full, and full stomachs, respectively.

## 3. Results

### 3.1. Gross Morphological Description

**Figure 2:**
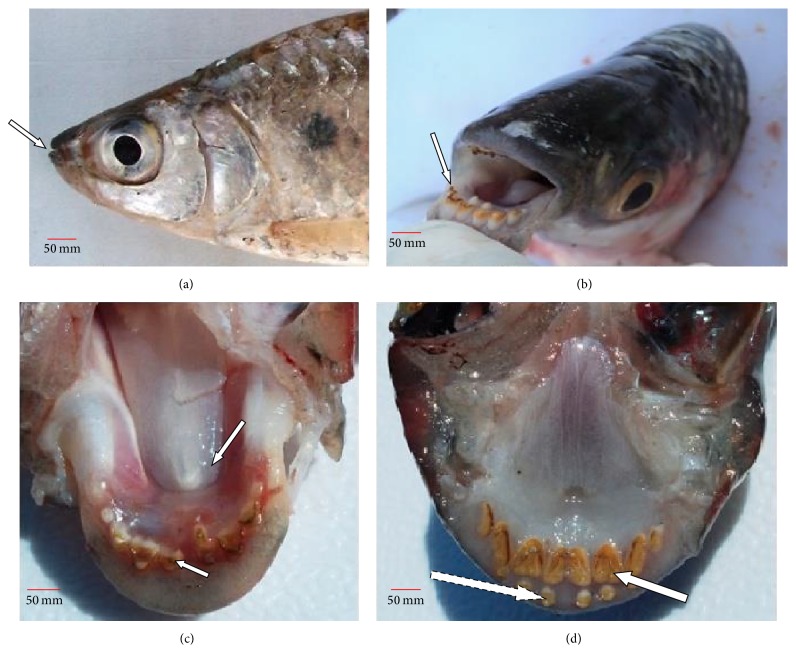
Gross morphology: (a) terminal mouth (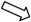), (b) dentition (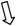), (c) tongue (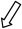) molar on the dentary (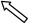) in the lower jaw, and (d) molar (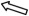) and conical teeth (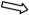) on the premaxilla in the upper jaw.

**Figure 3:**
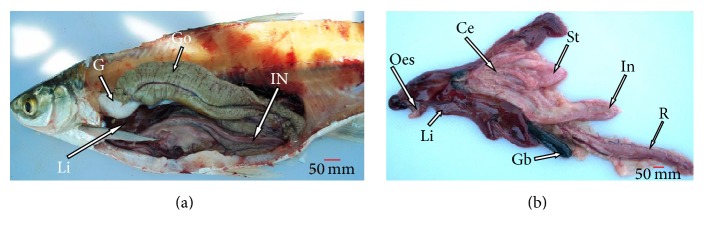
(a) Topography of the GIT and associated organs: gonad (Go), gas bladder (G), liver (Li), and intestine (IN). (b) Organs of the GIT: oesophagus (Oes), liver (Li), rectum (R), stomach (St), intestine (In), gall bladder (Gb), and caeca (Ce).

**Figure 4:**
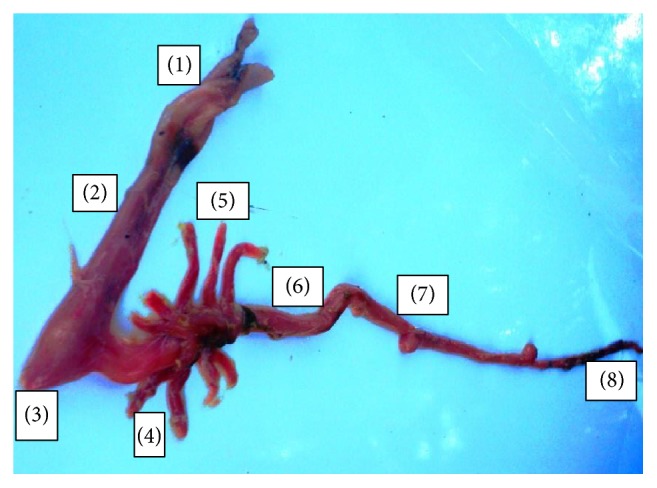
Structure of the digestive tract of* A. baremoze*. (1) Esophagus, (2) cardiac stomach, (3) fundic stomach, (4) pyloric stomach, (5) pyloric caeca, (6) anterior intestine, (7) mid intestine, and (8) posterior intestine/rectum.

*Alestes baremoze* has a fusiform shape typical of fast moving fish with relatively large scales. It has a bright silver colour which darkens on the dorsal surface with relatively large eyes, typical of characins. Gross morphological investigations revealed that the oral cavity opens through a fleshy-lipped terminal small mouth (Figure 2(a)). Inside the cavity was a soft textured tongue inclined to the lower jaw and large molars (Figure 2(b)) arranged in one raw (Figure 2(c)). The premaxilla had two teeth rows, one composed of conical teeth and the other composed of molars (Figure 2(d)). No valve was observed between the pharynx and the oesophagus. In the viscera (Figure 3(a)), the digestive system lay parallel to the gonads and the gas bladder. The short muscular oesophagus lay alongside the liver, posterior to the oral cavity, and joined the pharynx to the stomach. The muscular pouch-like stomach lay adjacent to the three-lobed liver and the anterior section of the intestine. The stomach was divided into the cardiac, fundic, and pyloric regions. The liver had two short lobes on the left, with one attached to the gall bladder and one longer lobe positioned to the right. A pyloric sphincter occurred between the pylorus section of the stomach and the duodenal section of the intestine, where nine-finger-like pyloric caeca were attached (Figure 3(b)). Posterior to the short duodenum was a looped intestine that lay alongside the gonads and gas bladder. The intestine had folds similar to those observed in the caeca (Figure 4). The intestinal diameter gently increased towards its hind section, ending into a narrow annual opening and the mean relative intestinal length was 1.2 ± 0.085 cm (Table 1).

### 3.2. Stomach Contents Analysis

Sixteen different prey categories were collected from the stomachs of 90 samples and recorded. Adult* A. baremoze* were found with stomachs predominantly containing insects (80.37%), with molluscs, crustaceans, and food items constituting lesser proportions (Table 2).

### 3.3. Histology

#### 3.3.1. Lip

**Figure 5:**
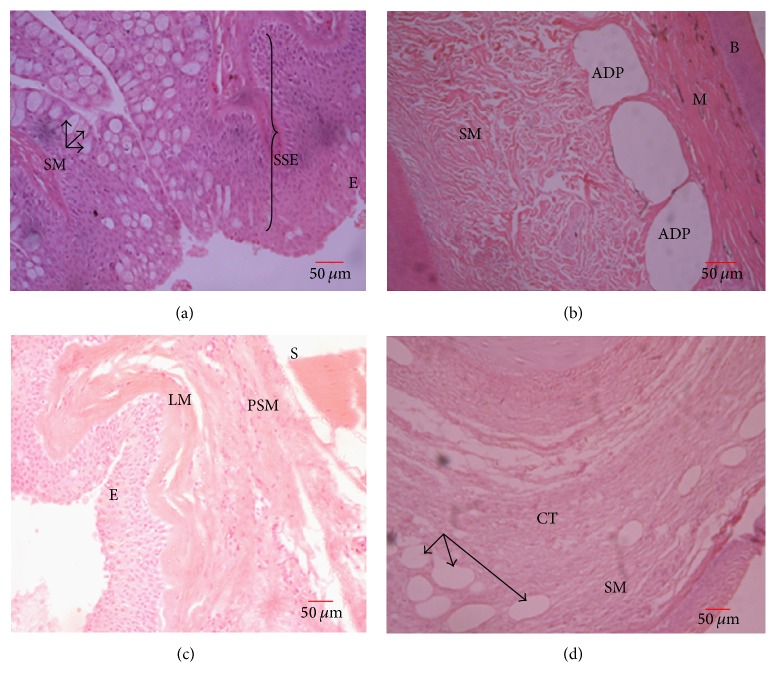
Oral cavity. (a) Lips: skeletal muscles (SM) and stratified squamous epithelium (SSE) with goblet cells (arrows). (b) Palate-roof of the oral cavity: epithelium (E), submucosa (SM) with irregular connective tissue, adipose tissue (ADP), skeletal muscle (M), and underlying bone (B). (c) Floor of the oral cavity: epithelium (E), laminar muscularis (LM), propria submucosa (PSM), and skeletal muscle (SM). (d) Tongue: adipose tissue (arrows), skeletal muscle (SM), connective tissue (CT), and underlying cartilage (C).

The lip surface was lined with a stratified squamous epithelium and numerous mucous secreting cells. Underlying this was cartilaginous and skeletal muscle. No taste buds were observed in the lip (Figure 5(a)).

#### 3.3.2. Palate

The roof of the oral cavity was formed by a palate lined by a stratified squamous epithelium with numerous epithelial folds (Figure 5(b)). Below this was the lamina muscularis which formed a demarcation between the epithelial layer and the propria submucosa. The propria submucosa of the palate was made of dense irregular connective tissue. Beneath this underlay cartilage and bone (Figure 5(c)).

#### 3.3.3. Tongue

The tongue was lined by a stratified squamous epithelium on the dorsum, with numerous lymphocytes-cell-like infiltrations (Figure 5(d)). The propria submucosa of the tongue was made of loose connective tissue with numerous adipose tissues. A thin layer of skeletal muscle, the lamina muscularis, separated the lamina epithelialis from the propria submucosa. The bulk of the tongue was shaped by underlying cartilage.

#### 3.3.4. Oesophagus

**Figure 6:**
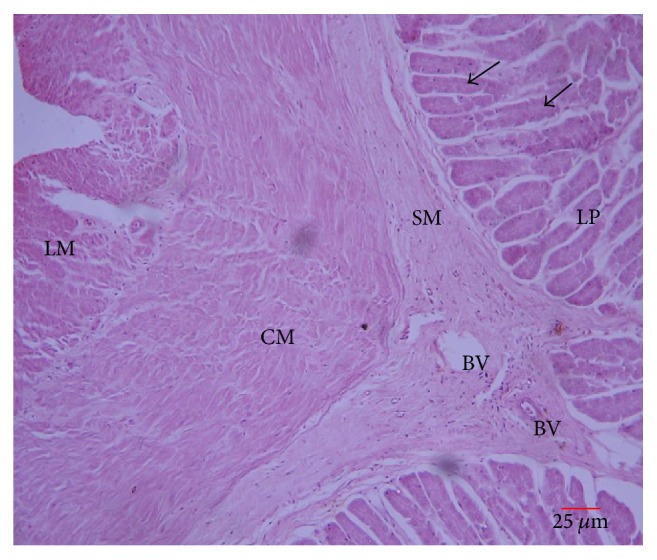
Transverse section of esophagus: lamina propria (LP), submucosa (SM), blood vessel (BV), inner circular muscle (CM), and outer longitudinal muscle (LM). Arrows: tubular glands.

*The* oesophagus was lined by a stratified squamous epithelium in the anterior portion which turned into a columnar epithelium towards the cardiac stomach (Figure 6). The lamina propria had numerous tubular glands throughout the entire length of the oesophagus. The mucosa was thrown into several folds supported by loose connective tissue from the submucosa. Numerous blood vessels were observed in the submucosa. The tunica muscularis had an inner circular muscle layer and an outer longitudinal skeletal muscle layer. Tunica muscularis turned from skeletal to smooth muscle towards the stomach.

#### 3.3.5. Stomach

**Figure 7:**
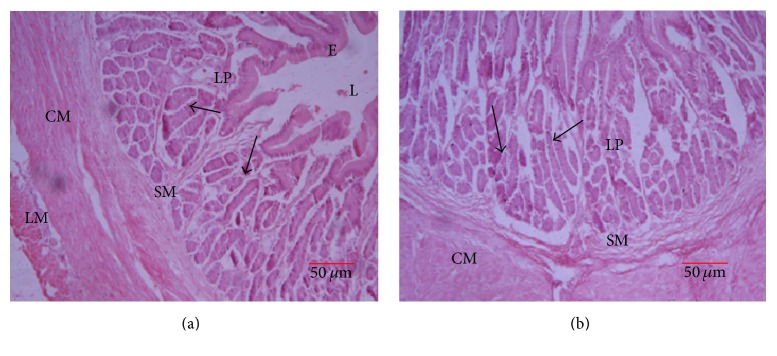
Transverse section through the stomach. (a) Cardiac stomach and (b) fundic stomach: lumen (L), epithelium (E), submucosa (SM), lamina propria (LP), circular muscle layer (CM), and longitudinal muscle (LM).

The stomach consisted of three distinct regions that were distinguished on the basis of glands in the lamina propria, the cardiac, fundic, and pyloric regions. In all the parts, the stomach was lined by a simple columnar epithelium. Laminar muscularis was not observed in the stomach. The lamina propria had numerous straight tubular glands (Figure 7(a)) that appeared shorter in the pyloric region as compared to the fundic and cardiac regions. The submucosa was made of loose connective tissue with numerous blood vessels. Tunica muscularis consisted of inner circular and outer longitudinal smooth muscle layers (Figure 7(b)). The longitudinal muscle layer was slightly thicker in the fundic region of the stomach. The serosa was a single layer of squamous cells beneath the muscularis.

#### 3.3.6. Liver

**Figure 8:**
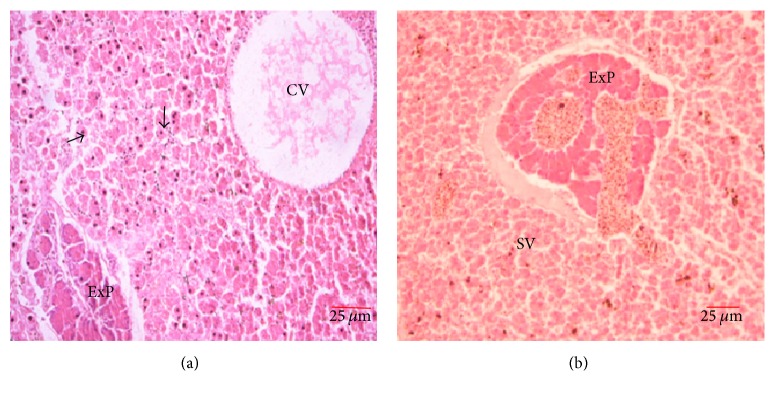
Liver: (a) CV, central vein, and (b) ExP, exocrine pancreas with secretory vesicles (SV), with hepatocytes arranged in irregular cords.

The liver was divided into lobules by connective tissue sheets with a central vein in the middle (Figure 8(a)). The hepatocytes were visible throughout the liver parenchyma arranged close to each other forming irregular cords. Throughout the liver lobules were scattered tubular acinar glands of the exocrine pancreas, forming the hepatopancreas and secretory vesicles (Figure 8(b)).

#### 3.3.7. Intestine

**Figure 9:**
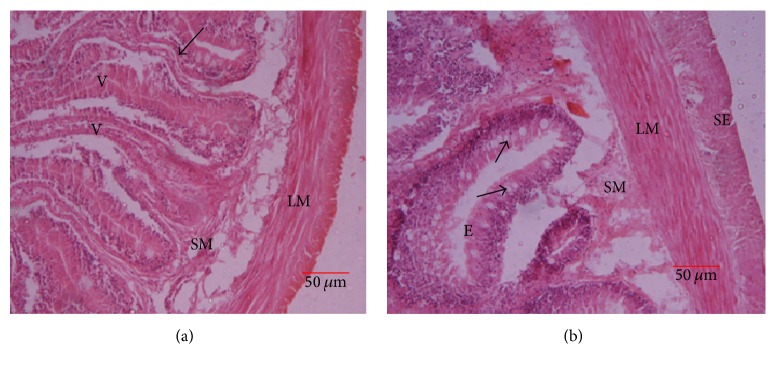
Intestine: E, epithelium, SM, submucosa, V, villi, SE, serosa, and LM, longitudinal muscle; arrows are goblet cells.

The mucosal layer of the intestine was thrown into folds (villi) of varying sizes (Figure 9(a)). Villi were tallest in the anterior part of the intestine. The mucosa was lined with a simple columnar epithelium with numerous lymphocytes cell-like infiltrations. Numerous goblet cells that increased in number towards the anal opening were seen in the lamina epithelialis. The submucosa was thin with its connective tissue extending the entire length of the villi. Tunica muscularis was made up of inner circular and outer longitudinal layer of smooth muscle (Figure 9(b)). The histological organization of the caeca was identical to that of the anterior and posterior intestine but with less villi and goblet cells.

## 4. Discussion

Histomorphological studies in fish have increasingly become important, given the growing interest in aquaculture [20], since the structure of the digestive system in fish indicates its feeding behaviour and provides insights into feeding strategies for cultured fish [21, 22]. Gross morphological investigations in this study revealed that the oral cavity in* A. baremoze* is composed of a small fleshy-lipped terminal mouth, with both conical and molariform dentitions. Similar mouth structures have been observed in* Cheilinus lunulatus* [23], with molariform and conical teeth common in* Clarias gariepinus* [24],* Herichthys minckleyi* [25],* Aphanius persicus* [26], and* Chrysobrycon eliasi* [27]. Terminal mouths in fish have been related to chasing and capture of small food items [28], while the presence of teeth on the jaws enables the predator to hold and grasp prey items [29]. Similarly, conical dentitions have been associated with soft bodied predators [30], while molariform dentations have been associated with mollusc and crustacean feeders [23]. The presence of these two dentition types in* A. baremoze* suggests that this fish feeds on a variety of food items but mainly of animal origin. Histological investigations further revealed the presence of several goblet cells in the epithelium lining the oral cavity, several skeletal muscles, adipose tissue, and an underlying cartilage in the cavity. Similar observations have been reported in* Oreochromis niloticus* and* Clarias gariepinus* [31], where presence of mucus secreting glands was suggested to imply secretion of copious amounts of mucus needed to lubricate the oral cavity to ease swallowing. Meanwhile the presence of muscle networks and adipose tissue suggests ease of mouth movement during mastication and a cushion of fat cells to act as a shock absorber during food acquisition and swallowing.

The oesophagus in* A. baremoze* in this study is short and has numerous tubular glands located in the lamina propria. The mucosa is also greatly folded, while the tunica muscularis is well developed with inner circular muscles and outer longitudinal skeletal muscles. Similar reports have been given in other fishes such as* Clarias gariepinus* and* Ctenopharyngodon idella* [32]. All these studies conclude that the numerous mucosal folds provide for increased surface area for chemical activity. Similarly, several tubular glands were also observed in the lamina propria of the oesophagus in* A. baremoze *in this study. This therefore suggests that chemical digestion in* A. baremoze *begins in the oesophagus.

Study results reveal the presence of a pouch-like stomach in* A. baremoze,* whose lamina propria had numerous straight tubular glands, as has been reported in* Esox lucius* [33, 34] and* Lates niloticus* [35]. In these fishes, the presence of a pouch-shaped stomach has been related to large feed quantity intake, which is slowly digested by chemical substances released from the tubular glands. This could be the mechanism of digestion also occurring in* A. baremoze.*

Morphological observations indicated that the liver in* A. baremoze* is three-lobed and lies adjacent to the oesophagus, stomach, and the anterior section of the intestine. Histologically, the liver parenchyma contained tubular acinar glands which constituted the exocrine pancreas forming the hepatopancreas. This arrangement is similar to what was observed in several fish and freshwater bivalves [17, 36]. However, the hepatopancreas is not observed in higher vertebrates [37].

Similarly, observations made in this study reveal that* A. baremoze* has a nine-fingered caeca and a long tubular intestine lying alongside the gonads. Multifingered caeca have been reported in several other fishes and have been proposed to be important in chemical digestion [38], absorption [39], osmoregulation [40], food storage, lipid absorption, and optimising pH for digestion [41]. Histologically, the organisation of the intestine did not significantly differ from that of the caeca. The arrangement of this structure is in agreement with findings by [42] and is suitable for absorbing digested food [43].

The RGL of* A. baremoze* in this study was 1.2 ± 0.085 (Table 1). The relationship between RGL and trophic levels in fishes has been documented [15]. This relationship is believed to reflect the digestive processing time required for different food material. Herbivorous fish species, for instance, tend to have higher RGL compared to omnivorous and carnivorous fishes due to the fibrous plant material they ingest, which requires longer digestion time. Although the RGL varies with fish species and growth stages, [16] categorises fish as either carnivorous (RGL = 0.6–0.8), omnivorous (RGL = 0.8–1.0), or herbivorous (RGL = 2.5–16.4). This suggests that* A. baremoze* is an omnivorous fish, a situation confirmed by observations made from its stomach contents (Table 2), which were seen to cut across plant and animal material. According to [1],* A. baremoze* has a flexible diet, shifting from zooplankton to zoobenthos, detritus, and macrophytes as plankton densities decline. Findings reported by [6] on food web structure in Lake Albert suggest that ontogenetic shifts occurred in the diet of* A. baremoze* and this was partly driven by structural changes in feeding apparatus and morphology that opened up new and unexploited feeding opportunities. Similarly, size specific shifts in food or habitat type have been documented in many species [44, 45]. Previous studies related to feeding ecology on one of the closest relatives,* Alestes nurse* in the Alestidae family, by [44] indicated that* A. nurse* was observed to change its food diet from phytoplankton and small zooplankton during their premetamorphosis stages to larger zooplankton. The observed dietary shifts observed in* A. baremoze* suggest that this fish can survive on a variety of food items and this makes it a good aquaculture candidate with ability to survive on formulated diets given that these are developed from a variety of raw materials both of plan and animal origin.

## 5. Conclusion

Histomorphological observations and stomach content analysis performed in this study suggest that* A. baremoze* has morphological adaptations for omnivory. The molariform dentation observed in the adult fish also helps them to feed on hard bodied organisms. Further investigations to assess the ability of* A. baremoze* to digest formulated diets will be useful.

## Figures and Tables

**Table 1:** Relative gut length of *A. baremoze *collected from Lake Albert, Uganda.

Month	Average body length (cm)	Average gut length (cm)	Sample number	Average relative gut length
April	42.5	53.8	10	1.3
May	42.5	51.1	20	1.2
June	42	49.7	10	1.2
July	37.7	51.9	5	1.4
August	39.5	53.3	7	1.3
September	41.8	46.3	11	1.1
October	38.8	47.1	8	1.2
November	42.5	49.8	10	1.2
December	40.3	50	9	1.2

Average RGL for all samples (April to December) = 1.2 ± 0.085.

**Table 2:** Point assessment percentages of various categories of food items in the stomachs of *Alestes baremoze* collected from Lake Albert, Uganda, during the year 2016.

Category	Food items	Point assessment percentages
Insecta	Remains of winged insect	90
	Dragonflies	80
	Ephemeroptera (nymph)	77
	Adult grasshoppers	74.5

Mollusca	Gastropoda	47.5

Crustacea	Ostracoda	45.3
	*Daphnia *	27
	Shrimps	27
	*Cyclops*	17
	*Moina *	11

Other food items	Detritus	22.9
	Phytoplankton (*Microcystis* and *Chlorella*)	2.6
	Eggs	2.58
	Fish scales	2.5
	Small stones	2.1
	Round worms	0.5
